# Perceived Controlling Behaviors of Physical Education Teachers and Objectively Measured Leisure-Time Physical Activity in Adolescents

**DOI:** 10.3390/ijerph16152709

**Published:** 2019-07-30

**Authors:** Andre Koka, Henri Tilga, Hanna Kalajas-Tilga, Vello Hein, Lennart Raudsepp

**Affiliations:** Institute of Sport Sciences and Physiotherapy, Faculty of Medicine, University of Tartu, Ujula 4 str., 51008 Tartu, Estonia

**Keywords:** controlling teaching, hierarchical model of intrinsic and extrinsic motivation, need frustration, moderate-to-vigorous physical activity, motivational regulations, self-determination theory

## Abstract

The facilitative role of perceived autonomy support from physical education (PE) teachers on adolescents’ leisure-time physical activity (PA) has been supported. This study aimed to examine the mechanism by which perceived controlling behaviors from PE teachers relate to adolescents’ objectively measured leisure-time PA. In a three-wave prospective study, a total of 159 students (64 boys) aged 11 to 19 years old (*M*_age_ = 14.94 years; *SD* = 2.11) completed measures of perceived controlling behavior, frustration of the basic psychological needs, and motivational regulations in PE. One week later, motivational regulations towards leisure-time moderate-to-vigorous PA (MVPA) were assessed. Five weeks later, MVPA was measured using accelerometers (ActiGraph GT3X) during seven consecutive days. Structural equation modeling analysis indicated that perceived controlling behavior predicted students’ frustration of basic psychological needs in PE. The competence need frustration in PE had a negative direct effect on objectively measured MVPA. A significant indirect effect supported the mediating role of competence frustration in PE in the relation between perceived controlling behavior from PE teachers and MVPA in adolescents. Findings suggest that future interventions striving to promote adolescents’ PA engagement in their leisure-time should focus on decreasing controlling behaviors from teachers in PE that may manipulate the teacher–student relation.

## 1. Introduction

Several systematic reviews support the importance of accumulating at least 60 min/day of moderate-to-vigorous physical activity (MVPA), as suggested by WHO [1], for the promotion of several aspects of health, as well as academic achievement in children and youth [2,3,4]. Although reported physical activity (PA) levels in children and youth may vary across countries [5], about 80% of adolescents worldwide do not meet the recommended guidelines [6]. Therefore, understanding the reasons why adolescents are not physically active is a crucial issue.

It has been recognized that physical education (PE) is one of the primary contexts for shaping PA habits for children and youth [7]. Research suggests that students’ perceptions of need-supportive teaching behaviors from their PE teachers are related positively to autonomous motivation for PE [8,9] and subsequently to adaptive outcomes, including higher levels of PA outside school measured via self-reporting [10,11] or objective measures [12,13,14,15]. In contrast, studies suggest that students’ perceptions of controlling teaching behaviors from their PE teachers are related to higher levels of controlled motivation and amotivation for PE [9,16,17] and to subsequent maladaptive outcomes, including higher levels of subjective ill-being [18]. There are only a few studies which demonstrate the unfavorable role of perceived controlling behaviors from PE teachers on self-reported leisure-time PA of adolescents [19,20]. According to Self-Determination Theory (SDT) [21,22] and Vallerand’s [23,24] Hierarchical Model of Intrinsic and Extrinsic Motivation (HMIEM), the current study was aimed to understand the mechanism by which perceived controlling behaviors from PE teachers are related to objectively measured PA of adolescents in their leisure-time.

Teachers are perceived as controlling when they use pressuring strategies to assure students comply and thereby, ignore their opinions [25]. According to SDT, the controlling interpersonal behavior by significant others (e.g., teachers, parents, coaches, etc.) could be expressed in two ways: in an externally controlling and in an internally controlling way [26,27,28]. Externally controlling behaviors involve external contingencies, such as reward and/or punishment, and these behaviors are often visible and overt [26]. For example, derived from previous work conducted in a coaching setting [29,30], Hein et al. [31] identified intimidating behavior and controlling use of praise as controlling strategies of the PE teacher that foster external regulation for students to behave in certain ways. Accordingly, intimidating behavior characterizes a PE teacher who uses the threat of punishment to motivate their pupils to work harder. An example of controlling use of praise is when a PE teacher uses positive feedback merely to direct students’ future behavior, instead of providing information regarding the present performance [31]. On the contrary, internally controlling behaviors are generally activated in subtle and covert ways and are meant to get students to coerce themselves by appealing to their feelings of anxiety, shame, and guilt [27]. For instance, teachers may use negative conditional regard, in which they provide less attention and affection when the student did not perform well or behaved as they were expected [16,31,32].

According to SDT [22,33], controlling teaching behaviors have a negative impact because such behaviors do not enable students to experience the satisfaction of basic psychological needs for autonomy (i.e., the need to perceive choice and responsibility in one’s action), competence (i.e., the need to feel efficiency in achieving desired outcome), and relatedness (i.e., the need to be connected to and accepted by significant others). Research conducted in a PE context has consistently shown that students experience feelings of need frustration in a controlling teaching environment [9,16,18,31,34,35]. Satisfaction versus frustration of these needs is assumed to determine the quality of motivation people will experience when engaging in a particular activity [33].

SDT distinguishes motivation regarding its quality, ranging from autonomous motivation (i.e., intrinsic motivation and identified regulation), over controlled motivation (i.e., introjected and external regulation), to amotivation (i.e., a lack of motivation) [33]. To illustrate, an autonomously motivated students partake in PE because they enjoy the fun derived from it, indicating intrinsic motivation, or because they realize that participating in PE is important for developing new sports skills, reflecting identified regulation. Research conducted in PE has shown that autonomous forms of motivation are associated with a number of adaptive outcomes including positive affect, preference to attempt challenging tasks, greater concentration and effort, and better grades [36,37,38,39]. In addition, higher levels of intrinsic motivation for PE has shown to be related to higher levels of objectively measured daily MVPA in adolescents [15]. On the contrary, a student who is controlled takes part in PE to receive social acceptance or to avoid internal pressures, indicating introjected regulation, or to prevent confrontation with the teacher, reflecting external regulation. Previous research has shown that controlled forms of motivation and amotivation for PE is associated with maladaptive outcomes, including boredom and unhappiness, decreased effort, and lower grades [36,37,39,40]. SDT-based studies in PE have indicated that perceived controlling behaviors from teachers are related to controlled forms of motivation and amotivation because they frustrate students’ basic psychological needs [9,16].

There is sufficient empirical evidence showing that students’ motivation experienced in a PE context is related to motivation towards PA outside of school, that is, in a leisure-time context [10,11]. Specifically, autonomous forms (i.e., intrinsic motivation and identified regulations) and controlled forms of motivation (i.e., introjected and external regulations) in PE were related to their respective forms of motivation in a leisure-time PA context [41]. The mechanism for this trans-contextual transfer of motivation is derived from the Vallerand’s [23,24] HMIEM, according to which reciprocal relationships might occur between motivation across distinct but similar contexts. The HMIEM has been proposed to be complementary to SDT, in that promoting or supporting individual’s behavior, such as PA, in an educational context (e.g., PE context) can lead to engagement in analogous activities in a different context. Instead, if the educational context is controlling rather than autonomy-supportive, compensatory behaviors can result [24]. It has been demonstrated that both intrinsic motivation and identified regulation for leisure-time PA were positive, whereas introjected regulation and external regulations were negatively associated with objectively measured MVPA in adolescents [42]. Only intrinsic motivation and external regulation for leisure-time PA, however, had statistically significant and inverse relationships with objectively measured MVPA.

To date, only a few studies [19,20] have investigated the role of perceived controlling behaviors from PE teachers on leisure-time PA of adolescents from a perspective of SDT. For example, students who were clustered as less self-determined towards PE reported significantly higher levels of teachers’ perceived controlling behavior and reported lower levels of psychological needs satisfaction, PA intentions, and self-reported leisure-time PA than students who were clustered as self-determined towards PE [20]. Results of Moreno-Murcia et al. [19] demonstrated that students’ perceptions of controlling behavior from PE teachers were negatively associated with students’ global intrinsic motivation. The global intrinsic motivation, in turn, positively predicted the perceived importance of the PE, which explained PA intentions and subsequent levels of self-reported PA. Although there are limitations to all PA measures, the present investigation was designed to examine the mechanism by which perceived controlling behaviors from PE teachers are related to objectively measured leisure-time MVPA in adolescents.

**Figure 1:**
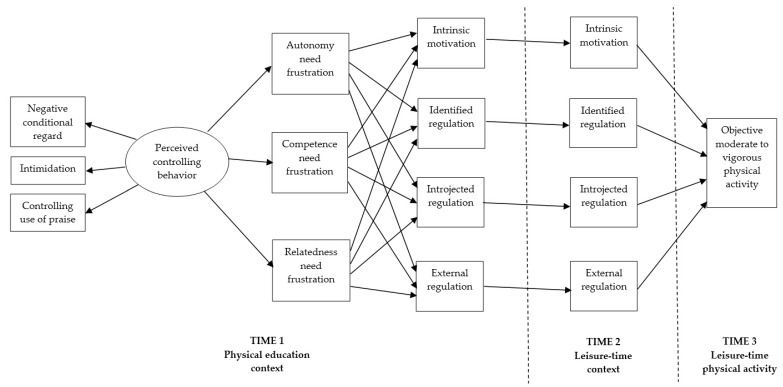
The hypothesized model. For clarity, the paths from past physical activity behavior to endogenous variables and error covariances among need frustration variables as well as different motivational regulation variables in physical education and leisure-time contexts are not depicted.

In line with SDT, HMIEM, and findings from previous research, the hypothesized model, depicted in Figure 1, was proposed. Specifically, it was expected that students’ perceptions of PE teachers’ controlling behavior would contribute to higher levels of perceived frustration of the need for autonomy, competence, and relatedness, which, in turn, would contribute to lower levels of intrinsic motivation and identified regulation, but higher levels of introjected regulation and external regulation in PE. Each motivational regulation was anticipated to demonstrate significant within-construct, trans-contextual regression from the PE context to the leisure-time context. In turn, whereas intrinsic motivation and identified regulation for leisure-time PA was hypothesized to result in higher levels of objectively measured MVPA, introjected regulation and external regulation was hypothesized to result in lower levels of objectively measured MVPA. The negative effect of perceived controlling behavior from PE teachers on adolescents’ objectively measured MVPA was hypothesized to be indirect through the perceived frustration of psychological needs and controlled forms of motivational regulations (i.e., introjected and external regulations) in PE and leisure-time contexts. Finally, according to previous work in a PE and leisure-time PA contexts [41,43,44], the effect of past PA behavior on the relations posited in the hypothesized model was controlled for by specifying paths from past PA behavior to all variables.

## 2. Materials and Methods

### 2.1. Participants

Complete data across the three points of data collection were acquired from 159 students aged 11 to 19 years (*M*_age_ = 14.94 years; *SD* = 2.11; 64 boys and 95 girls) who were recruited from an initial sample of 285 participants from eight state schools. Attrition was attributed mainly to not completing questionnaires at time 2 (*n* = 22) due to absences of students on the testing day (e.g., due to illness) or not providing adequate accelerometer data at time 3 (*n* = 104).

### 2.2. Procedure

Before the study, the institutional, parental, and student permission was obtained. In keeping with previous research conducted in a PE and leisure-time PA contexts [13,41,43,44], this study adopted a multi-wave design. During the first wave of data collection (Time 1), students completed a multi-section instrument assessing the perceptions of their teachers’ controlling behavior, perceived frustration of the needs for competence, autonomy, and relatedness, and different forms of motivational regulations in a PE context. One week later, a questionnaire administered in a second-wave of data collection (Time 2) measured different forms of motivational regulations towards leisure-time PA, as well as the incidence of leisure-time PA in the past 6 months. The one-week interval between the data collection of Time 1 and Time 2 is in line with previous studies [13,41,43,44] and was used to reduce the common method variance related to the use of similar measures (i.e., different forms of motivational regulations towards PE and leisure-time PA). Five weeks after the second-wave of data collection (i.e., Time 3), students were asked to wear the accelerometer for seven consecutive days preceded by the written and verbal instruction regarding the placement and wearing the unit. A five-week period between the data collection of Time 2 and Time 3 was used as it permits a longer range prediction of PA behavior, as suggested by Hagger et al. [43].

All of the questionnaires were completed in a quiet classroom condition and anonymously to preserve confidentiality. Prospective responses to the questionnaires were matched by using the participant’s gender and date of birth. To ensure the accelerometer data were matched to the responses to questionnaires, a matching numerical code was assigned to each participant and accelerometer. All waves of data collections were carried out between October 2018 and December 2018. The design and procedures of the present study were approved by the local university ethical committee.

### 2.3. Measures

#### 2.3.1. Teachers’ Perceived Controlling Behavior

The Controlling Coach Behaviors Scale (CCBS) [30], modified to the PE context [31], was used to assess students’ perceptions of controlling behaviors from their teachers. Students were asked to indicate on a 7-point Likert scale, ranging from 1 (strongly disagree) to 7 (strongly agree), how true each statement was with regard to the PE teacher who taught them. Three three-item subscales (negative conditional regard, controlling use of praise, and intimidation), taken from the PE-modified version of the CCBS, were used for the purpose of the present study. Example items included: “My PE teacher only uses praise so that I stay focused on tasks during lesson” (controlling use of praise); “My PE teacher is less friendly with me if I don't make the effort to see things his/her way” (negative conditional regard); and “My PE teacher intimidates me into doing the things that he/she wants me to do” (intimidation). The subscale of excessive controlling behavior (e.g., “My PE teacher tries to interfere in aspects of my activities outside of school”) was excluded from the present study as it has shown to be irrelevant to the PE context [31]. Previous PE-based studies have provided evidence for the reliability of scores using Cronbach’s alphas and factorial structure of the scale [19,35,45].

#### 2.3.2. Perceived Frustration of the Basic Psychological Needs

The respective subscales from the PE-adapted Basic Psychological Need Satisfaction and Frustration Scale (BPNSFS) [9] were used to assess students’ perceptions of frustration of the needs for autonomy, competence, and relatedness. Items were presented with a common stem (“During the PE lesson…”), followed by items such as “…I felt pressured to do too many exercises” (frustration of the need for autonomy), “…I felt insecure about my abilities” (frustration of the need for competence), and “…I felt excluded from the group I want to belong to” (frustration of the need for relatedness). Each subscale consists of four items. Responses to the items were provided on a 7-point Likert scale ranging from 1 (strongly disagree) to 7 (strongly agree). Previous PE-based research [9,35] has provided evidence for the reliability and factorial structure of the scale.

#### 2.3.3. Motivational Regulations in Physical Education Context

The Perceived Locus of Causality (PLOC) scale [46] was used to assess different types of motivational regulations of students in PE. Participants were asked to respond to the items using the stem: “I take part in PE...”, followed by different reasons: intrinsic motivation (e.g., “...because I enjoy PE”); identified regulation (e.g., “...because I value the benefits of PE”); introjected regulation (e.g., “...because I will feel bad about myself if I did not”); and external regulation (e.g., “...because I will get into trouble if I do not”). All subscales consist of four items, and students were instructed to respond on a 7-point Likert scale ranging from 1 (strongly disagree) to 7 (strongly agree). Previous research with school students has supported the reliability and validity of the PLOC scale [13,47,48].

#### 2.3.4. Motivational Regulations in Leisure-Time Physical Activity Context

The Behavioral Regulations in Exercise Questionnaire (BREQ) [49] was used to assess different forms of motivational regulations of students in a leisure-time PA context. Students were asked to respond to the items using the common stem: “I take part in active sports and/or moderate-to-vigorous physical activities in my spare time...”, followed by different reasons: intrinsic motivation (e.g., “...because I enjoy doing physical activity”); identified regulation (e.g., “...because it is important to me to do physical activities”); introjected regulation (e.g., “…because I will feel guilty if I do not”); and external regulation (e.g., “...because other people I know will not be pleased with me if I do not do physical activity). All subscales consist of four items. Responses were assessed on a 7-point Likert scale ranging from 1 (strongly disagree) to 7 (strongly agree). Previous research has supported the tenability of the BREQ to similar age student samples [41,43,44].

#### 2.3.5. Objectively Measured Leisure-Time Moderate-To-Vigorous Physical Activity

Students’ MVPA was monitored for seven consecutive days with accelerometer Actigraph GT3X (ActiGraph LLC, Pensacola, FL, USA) using the sampling interval of 15 seconds. Participants were guided to wear the accelerometer on their waist and to remove it only for sleeping and water-based activities, such as swimming or bathing, while retaining their usual activity. The collected data was downloaded and processed using ActiLife software 6.13.3 (ActiGraph LLC, Pensacola, FL, USA). Data were considered valid if at least four days, included at least one weekend day, with a minimum of 600 min of wearing time was reported. Zero counts of consecutive 60 min were considered as non-wear time. The time spent in MVPA was calculated based on Evenson et al. [50] cut-points of ≥2296 activity counts per minute. For each participant, the average minutes of MVPA over valid days were calculated. During the wearing period, students were also asked to fill the accelerometer diary every day and write down the beginning and end time for sleep, school, and PE lessons. The information from students’ diaries was used to set time filters to obtain the PA data at leisure-time.

#### 2.3.6. Past Moderate-To-Vigorous Physical Activity Behavior

In accordance with previous studies [41,43,44,51], students’ past MVPA behavior was assessed using one item asking the participants to report how often they had been doing active sports and/or moderate-to-vigorous physical activities during the last 6 months. Responses were assessed on a 6-point scale anchored by 1 (not at all) to 6 (most of the days per week). Before the administration of this behavioral measure, participants were provided with the definition of MVPA. 

### 2.4. Data Analysis 

The data were analyzed using SPSS 23.0 (IBM corp., Armonk, NY, USA) for Windows and IBM SPSS AMOS 23.0 (IBM corp., Armonk, NY, USA). Descriptive statistics, including means and standard deviations, were computed for each study variable. Using dummy-coding (stay vs. dropout), depending on the distribution of the data, the independent samples *t*-test or Mann–Whitney *U*-test was carried out on the mean scores of all the study variables to examine for possible differences between students who completed the study measures at all time-points and students who dropped out from the study following the completion of Time 1 measures. The internal consistency of each scale was assessed using Cronbach’s alpha value, and zero-order correlations among study variables were computed.

A structural equation modeling (SEM) with maximum likelihood estimation method was used to examine the hypothesized relations among study variables. In the hypothesized model, depicted in Figure 1, non-latent composite variables, except for the latent perceived controlling behavior that was indicated by three composite variables for negative conditional regard, controlling use of praise, and intimidation, were used. Several indices were used to assess the adequacy of the fit of the hypothesized model to the data: Comparative Fit Index (CFI), Non-Normed Fit Index (NNFI), and Root Mean Squared Error of Approximation (RMSEA) with its 90% confidence intervals (CI_90_). A model that fits the data well is indicated when values for CFI and NNFI are ≥0.95 [52]. The values ≤0.06 for RMSEA indicate good fit, with values ≤0.10 for its upper limit of CI_90_ [53]. The SEM analysis was complemented by bootstrapping analysis, as recommended by Preacher and Hayes [54], to determine the standardized estimates and significance levels for indirect effects in the hypothesized model. The indirect effect was considered statistically significant if its 95% confidence intervals did not include the zero. 

## 3. Results

### 3.1. Drop-Out Analyses of the Participants

The independent samples *t*-test or Mann–Whitney *U*-test revealed that students who dropped out from the study following the completion of Time 1 measures perceived their teachers to exhibit significantly higher negative conditional regard (*M* = 2.98, *SD* = 1.43 vs. *M* = 2.59, *SD* = 1.27, *t* = 2.30, *p* < 0.05) and intimidation behavior (*M* = 2.07, *SD* = 1.20 vs. *M* = 1.72, *SD* = 0.96, *U* = 6795, *z* = −2.51, *p* < 0.05), and scored significantly higher on amotivation towards PE (*M* = 2.52, *SD* = 1.41 vs. *M* = 2.09, *SD* = 1.32, *t* = 2.48, *p* < 0.05) than students who retained for analyses. There were no significant differences either in age (*t* = 1.34, *p* > 0.05) or distribution of gender (χ^2^ = 2.92, *p* > 0.05) between students who dropped out from the study and those who retained for analyses.

### 3.2. Descriptive Statistics

Descriptive statistics, Cronbach’s alpha coefficients, and correlations among study variables for those students (*n* = 159) who completed the study measures at all time points are presented in Table 1. Results showed that mean scores were slightly below the midpoint for all variables except intrinsic motivation and identified regulation in both PE and leisure-time contexts, introjected and external regulation in PE, and self-reported past leisure-time PA. The Cronbach’s alpha coefficients for all the subscales exceeded the traditional criterion for an acceptable level of internal consistency (i.e., α ≥ 0.70) [55], except negative conditional regard, intimidation, controlling use of praise, autonomy need frustration, and introjected regulation in PE.

The correlational analysis revealed that perceived controlling behaviors had significant and positive correlations with perceived frustration of all three psychological needs and external regulation in PE. In addition, psychological needs frustration in PE were significantly and positively correlated with external regulation in PE, whereas significantly and negatively with intrinsic motivation in PE and leisure-time. Further, correlations supported strong relationships between intrinsic motivation, identified, introjected, and external regulation in a PE context with the same motivational regulation types in a leisure-time context. Finally, frustration of the need for competence in PE had a significant and negative relationship with objectively measured MVPA, whereas intrinsic motivation, identified and introjected regulation for leisure-time PA had a significant and positive relationship with objectively measured MVPA.

### 3.3. Structural Equation Modelling

The hypothesized model exhibited adequate fit to the data, χ^2^ = 126.80, df = 65; CFI = 0.94; RMSEA = 0.077, CI_90_ for RMSEA range = 0.057–0.097. However, the modification indices suggested to add one path, namely competence need frustration → objectively measured MVPA. This slightly amended model was reanalyzed and resulted in a significant change in the χ^2^ value compared with the initial model (Δχ^2^ = 6.58, Δdf = 1, *p* < 0.01). This indicated that the added competence need frustration → objectively measured MVPA path was meaningful. The new results revealed a marginal improvement in the fit of the model to the data, χ^2^ = 119.80, df = 64; CFI = 0.95; RMSEA = 0.074, CI_90_ for RMSEA range = 0.053–0.095.

**Figure 2:**
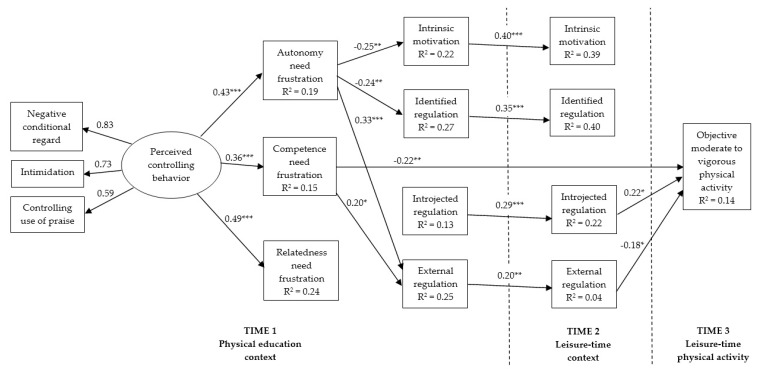
Objectively measured leisure-time moderate-to-vigorous physical activity in adolescents predicted by teachers’ perceived controlling behavior and basic psychological needs frustration in PE, and different motivational regulations in PE and leisure-time context. For clarity, only significant paths are presented. In addition, the effects of past physical activity behavior on endogenous variables and error covariances among need frustration variables as well as different motivational regulation variables in both PE and leisure-time contexts are not depicted. Past physical activity behavior exerted significant direct relationships with competence need frustration (−0.17 *), intrinsic motivation in PE (0.31 ***), identified regulation in PE (0.37 ***), introjected regulation in PE (0.33 ***), intrinsic motivation in leisure-time (0.36 ***), identified regulation in leisure-time (0.41 ***), and introjected regulation in leisure-time (0.29 ***). * *p* < 0.05, ** *p* < 0.01, *** *p* < 0.001.

The standardized path coefficients for statistically significant free parameters for the final model are shown in Figure 2. Results revealed that perceived controlling behavior was positively related to autonomy, competence, and relatedness need frustration in PE. Autonomy need frustration was negatively related to intrinsic motivation and identified regulation in PE, whereas both competence and autonomy frustration were positively related to external regulation in PE. Competence need frustration in PE had also a direct and negative relation with objectively measured MVPA. All motivational regulations in PE were significantly related to their respective motivational regulations in leisure-time. Whereas, introjected regulation in leisure-time was positively related to students’ objectively measured MVPA, the external regulation was negatively related to MVPA. The model accounted for 14% of the variance in students’ objectively measured MVPA.

#### 3.3.1. Indirect Effects

The standardized parameter estimates of indirect effects along with their 95% lower and upper limits of bootstrapped-generated bias-corrected confidence intervals are presented in Table 2. The results indicated that perceived controlling behavior had a positive indirect effect on external regulation in both PE and leisure-time, whereas it had a negative indirect effect on intrinsic motivation in both PE and leisure-time and on objectively measured MVPA. Autonomy need frustration in PE had a positive indirect effect on external regulation in leisure-time, whereas it had a negative indirect effect on both intrinsic motivation and identified regulation in leisure-time. Competence need frustration in PE had a positive indirect effect on external regulation in leisure-time. The positive indirect effect was shown for introjected regulation in PE on objectively measured MVPA, whereas a negative indirect effect was shown for external regulation in PE on objectively measured MVPA. Lastly, the positive indirect effect for relatedness need frustration on identified regulation in leisure-time approached the significance.

#### 3.3.2. Specific Indirect Effects

Based on the inspection of significant single paths shown in Figure 2, we estimated the significance of three specific indirect effects from perceived controlling behavior to objectively measured MVPA: (a) via the competence need frustration; (b) via the sequence of autonomy need frustration, external regulation in PE, and external regulation in leisure-time; and (c) via the sequence of competence need frustration, external regulation in PE, and external regulation in leisure-time. Results showed that the specific indirect effect of perceived controlling behavior on objectively measured MVPA via the competence need frustration was the strongest (B = −1.35; 95% CI = −3.14, −0.27; β = −0.08; *p* < 0.01). Specific indirect effects via the sequence of autonomy need frustration, external regulation in PE, and external regulation in leisure-time (B = −0.08; 95% CI = −0.32, −0.01; β = −0.01; *p* < 0.05) and via the sequence of competence need frustration, external regulation in PE, and external regulation in leisure-time (B = −0.04; 95% CI = −0.21, −0.00; β = −0.00; *p* < 0.05) were weak and similar, though marginally significant.

## 4. Discussion

The unique contribution of the present study is that it attempted to understand the mechanism of how students’ perceptions of teachers’ controlling behavior in a PE context relate to objectively measured MVPA in another, yet related context (i.e., leisure-time PA context). Results revealed that perceived controlling behavior from PE teachers had a detrimental effect on adolescents’ objectively measured MVPA because it led to higher levels of perceived frustration of their need for competence in PE classes. It was also found that higher levels of external regulation towards PE and leisure-time PA as a consequence of perceived frustration of the needs for competence and autonomy in PE, may contribute to the understanding of the unfavorable effect of teachers’ perceived controlling behavior on adolescents’ objectively measured MVPA.

Consistent with SDT [22,33] and our hypothesis, perceived controlling behavior from PE teachers was associated with higher perceived frustration of basic psychological needs of students, which, in turn, except relatedness need frustration, was related to higher levels of external regulation towards PE. Moreover, akin to the results of Bartholomew et al. [16], the emerged decrease in students’ intrinsic motivation and identified regulation towards PE is particularly worrying, given the corresponding increase in external regulation. Consistent with the past work [41] informed by the HMIEM [23,24], and supporting our hypothesis, students’ motivational regulations towards PE were significantly related to the respective motivational regulations in a leisure-time PA context. Importantly, indirect effects showed the significant decreasing effect of perceived controlling behavior from teachers on adolescents’ intrinsic motivation towards leisure-time PA, but the significant increasing effect on external regulation towards leisure-time PA. Collectively, these findings corroborate past PE-related studies documenting motivational disadvantages associated with being taught by controlling teachers [9,16,17,19,20].

Results revealed that perceived frustration of the need for competence in PE had a direct decreasing effect on adolescents’ objectively measured MVPA. This was the only posteriori effect included in the model based on modification indices. The central role of competence in PE on both self-reported and objectively measured leisure-time PA is consistent with previous research with adolescents [13,56]. While the latter studies highlighted the importance of satisfying the adolescents’ need for competence in PE, the present study adds empirical evidence to the extant literature by demonstrating the importance of avoiding the experience of competence need frustration in PE, if the aim is to promote leisure-time MVPA in adolescents. Moreover, the results of the tests of specific indirect effects showed that the indirect effect via the perceived frustration of the need for competence was the strongest in explaining the relationship between perceived controlling behavior and objectively measured MVPA. As a consequence of this finding, some suggestions for practicing PE teachers can be provided on which behaviors they should avoid that their students do not experience frustration of the need for competence in PE and thereby, do not keep away from PA in their leisure-time. PE teachers are encouraged to avoid behaviors, such as shouting at pupils in front of others to force them to do certain things (i.e., intimidating behavior); being less supportive to students if they have displeased him/her (i.e., negative conditional regard); and using praise only to make students stay focused on tasks (i.e., controlling use of praise). Such behaviors have been demonstrated to be associated with higher frustration and thwarting of the basic psychological needs of students in PE [16,31,34,35], which may have a detrimental effect on leisure-time MVPA.

This study found, consistent with our hypothesis and results of the previous study with adolescents [42], that external regulation for leisure-time PA was related to lower levels of objectively measured MVPA. This finding suggests that adolescents’ MVPA levels decrease as a function of increased belief that PA is something they just must do; otherwise, they will get into trouble. It is noteworthy, and contrary to our expectation, that introjected regulation for leisure-time PA was related to higher levels of objectively measured MVPA in adolescents. This finding, however, was not completely surprising as previous research has shown that introjected regulation, described as internalized social pressures to be active, may also become influential for some children [57,58]. Nevertheless, the positive association of introjected regulation with PA has shown to be short-term rather than long-term [59,60]. Therefore, a persistent emphasis on enjoyment (i.e., intrinsic motivation) and personal benefits (i.e., identified regulation) associated with PA has been suggested to avoid a dominant internalized social pressure to be physically active [61]. Interestingly, however, although significant zero-order correlations of intrinsic motivation and identified regulation towards leisure-time PA with objectively measured MVPA emerged in the present study, results of the SEM analysis revealed no unique effects of intrinsic motivation and identified regulation towards leisure-time PA on objectively measured MVPA. The findings suggest that intrinsic motivation and identified regulations as adaptive forms of motivational regulations failed to contribute significantly to objectively measured MVPA when examining associations between perceived controlling behavior and maladaptive motivational processes (e.g., frustration of psychological needs and maladaptive forms of motivational regulations) and adaptive outcomes, such as MVPA.

Although the current study provided unique information in terms of the mechanism by which perceived controlling behavior from teachers in a context of PE is associated with objectively measured MVPA of adolescents in a leisure-time context, this study is not without limitations. First, at the beginning of the study, the sample size was quite adequate. The high attrition across the three waves of data collection, however, is the main limitation of this study. In addition, the fact must also be acknowledged that students who dropped out from the study following the completion of Time 1 measures had significantly higher perceptions of intimidation and negative conditional regard from teachers, as well as higher levels of amotivation towards PE than students who retained for analyses. One may argue, therefore, that attrition might have influenced the results obtained in the current study. Second, although the experience of past PA behavior was taken into the account when testing the relations among study variables in the hypothesized model, the data related to the past PA behavior was obtained through adolescents’ self-reports, whereas the MVPA as a dependent variable was assessed using accelerometers. The discrepancy between the measurements of PA may be the cause of the non-significant effect of past PA on objectively measured MVPA in the SEM analysis. Third, the data about teachers’ behavior collected from students were entirely self-reports, thus limiting to obtain an accurate picture of teachers’ behavior. It has been recommended that ratings of external observers, in addition to students’ self-reports, should be taken into account to obtain a more accurate picture of teachers’ behavior exhibited in the classroom [9]. Forth, because of the sophisticated hypothesized model and relatively small final sample size, most of the variables were treated as composite variables rather than latent variables. Because of this, the effect size of the paths may have been decreased. Therefore, future studies would do well by replicating the present findings with a larger sample which would allow to use the latent variables and thus enable to account for measurement errors. Fifth, even though this study adopted a multi-wave design, the essence of the data, principally, is correlational, thus precluding the inference of causality. In addition, all variables were assessed at one time-point and, therefore, reciprocal relations between variables could not be tested. Finally, situation-specific beliefs about leisure-time PA behavior, specified in the Theory of Planned Behavior (TPB) [62], was not examined in the present study. Past studies have demonstrated that constructs from the TPB, such as attitude, perceived behavioral control, subjective norms, and intention towards PA, contribute to the explaining the relations between motivational processes in PE and leisure-time PA behavior [41,43,44,63,64]. Future studies examining the role of perceived controlling behavior from PE teachers on adolescents’ objectively measured MVPA would do well by including also the constructs from the TPB that might provide an additional explanation for the mechanism behind the relationship.

## 5. Conclusions

Despite the limitations, the present study revealed that the more students perceived their PE teachers as controlling when communicating with them in classes, the less they were physically active in their leisure-time. This is because controlling behaviors are likely to frustrate basic psychological needs, but particularly the need for competence, which may be one of the primary cause for adolescents to refrain from being physically active outside of school. Findings suggest that future interventions striving to promote adolescents’ motivation towards PA and PA behavior in leisure-time should focus on decreasing teachers’ controlling behaviors that may manipulate the teacher-student relation.

## Figures and Tables

**Table 1:** Descriptive statistics, Cronbach’s alpha coefficients, and correlations among study variables.

	Correlations															
Variable	1	2	3	4	5	6	7	8	9	10	11	12	13	14	15	16
1. Negative conditional regard	-															
2. Intimidation	0.62 **	-														
3. Controlling use of praise	0.49 **	0.40 **	-													
4. Autonomy need frustration	0.34 **	0.29 **	0.32 **	-												
5. Competence need frustration	0.31 **	0.23 **	0.21 **	0.66 **	-											
6. Relatedness need frustration	0.39 **	0.35 **	0.36 **	0.45 **	0.52 **	-										
7. Intrinsic motivation in PE	−0.02	−0.13	−0.03	−0.34 **	−0.31 **	−0.20 *	-									
8. Identified regulation in PE	0.01	−0.05	0.04	−0.32 **	−0.32 **	−0.06	0.81 **	-								
9. Introjected regulation in PE	0.09	0.17 *	0.10	0.04	−0.03	0.09	0.43 **	0.56 **	-							
10. External regulation in PE	0.37 **	0.40 **	0.30 **	0.46 **	0.42 **	0.28 **	−0.11	−0.01	0.44 **	-						
11. Intrinsic motivation in LT	−0.10	−0.13	−0.05	−0.25 **	−0.34 **	−0.23 **	0.53 **	0.46 **	0.19 *	−0.15	-					
12. Identified regulation in LT	0.01	0.04	0.01	−0.09	−0.21 **	−0.15	0.49 **	0.51 **	0.44 **	0.05	0.65 **	-				
13. Introjected regulation in LT	0.12	0.15	0.13	0.14	0.11	0.23 **	0.25 **	0.30 **	0.51 **	0.29 **	0.20 *	0.59 **	-			
14. External regulation in LT	0.15	0.20 *	0.21 **	0.13	0.20 *	0.29 **	0.08	0.18 *	0.32 **	0.29 **	−0.18 *	0.05	0.38 **	-		
15. Objective MVPA (min/day)	0.01	0.05	−0.09	−0.12	−0.23 **	−0.12	0.09	0.07	0.08	−0.01	0.22 **	0.21 **	0.16 *	−0.14	-	
16. Past LT physical activity	0.03	0.01	0.10	−0.08	−0.16	−0.05	0.34 **	0.40 **	0.33 **	0.04	0.50 **	0.55 **	0.37 **	0.03	0.21 **	-
Mean	2.56	1.72	3.30	3.13	3.02	2.10	5.27	5.23	3.96	4.21	5.27	4.38	2.93	2.19	38.75	3.77
SD	1.27	0.96	1.34	1.17	1.36	1.19	1.31	1.27	1.31	1.45	1.44	1.36	1.41	1.15	17.57	1.13
Cronbach alpha (α)	0.64	0.67	0.66	0.65	0.78	0.83	0.87	0.84	0.64	0.74	0.89	0.77	0.80	0.74	-	-

Notes: PE = physical education; LT = leisure-time; MVPA = moderate-to-vigorous physical activity. Variables from 1 to 14 were measured on a 7-point scale, whereas variable 16 was measured on a 6-point scale. * *p* < 0.05, ** *p* < 0.01.

**Table 2:** Standardized parameter estimates of indirect effects.

Parameter	β	95% CI	
		LL	UL
Perceived controlling behavior → Intrinsic motivation in PE	−0.15 ***	−0.26	−0.06
Perceived controlling behavior → Identified regulation in PE	−0.09	−0.20	0.01
Perceived controlling behavior → Introjected regulation in PE	−0.06	−0.02	0.15
Perceived controlling behavior → External regulation in PE	0.23 ***	0.12	0.36
Perceived controlling behavior → Intrinsic motivation in LT	−0.06 ***	−0.11	−0.02
Perceived controlling behavior → Identified regulation in LT	−0.03	−0.08	0.00
Perceived controlling behavior → Introjected regulation in LT	0.02	−0.00	0.16
Perceived controlling behavior → External regulation in LT	0.04 *	0.01	0.10
Perceived controlling behavior → Objectively measured MVPA	−0.09 **	−0.19	−0.02
Autonomy need frustration → Intrinsic motivation in LT	−0.10 **	−0.19	−0.03
Autonomy need frustration → Identified regulation in LT	−0.08 **	−0.17	−0.03
Autonomy need frustration → Introjected regulation in LT	0.02	−0.03	0.09
Autonomy need frustration → External regulation in LT	0.06 *	0.01	0.14
Autonomy need frustration → Objectively measured MVPA	−0.01	−0.04	0.02
Competence need frustration → Intrinsic motivation in LT	−0.03	−0.13	0.05
Competence need frustration → Identified regulation in LT	−0.06	−0.15	0.01
Competence need frustration → Introjected regulation in LT	−0.02	−0.10	0.04
Competence need frustration → External regulation in LT	0.04 *	0.00	0.11
Competence need frustration → Objectively measured MVPA	−0.01	−0.04	0.01
Relatedness need frustration → Intrinsic motivation in LT	−0.01	−0.09	0.06
Relatedness need frustration → Identified regulation in LT	0.06 ^a^	0.00	0.12
Relatedness need frustration → Introjected regulation in LT	0.04	−0.01	0.11
Relatedness need frustration → External regulation in LT	0.01	−0.03	0.05
Relatedness need frustration → Objectively measured MVPA	0.00	−0.02	0.03
Intrinsic motivation in PE → Objectively measured MVPA	0.03	−0.05	0.11
Identified regulation in PE → Objectively measured MVPA	−0.02	−0.12	0.06
Introjected regulation in PE → Objectively measured MVPA	0.06 *	0.01	0.15
External regulation in PE → Objectively measured MVPA	−0.04 *	−0.10	−0.01

Notes: β = standardized parameter estimate; 95% CI = 95% confidence intervals of parameter estimates; LL = Lower limit of 95% CI; UL = Upper limit of 95% CI; PE = physical education; LT = leisure-time; MVPA = moderate-to-vigorous physical activity. * *p* < 0.05, ** *p* < 0.01, *** *p* < 0.001, ^a^ *p* = 0.05.
